# Hepatocyte-Derived Igκ Exerts a Protective Effect against ConA-Induced Acute Liver Injury

**DOI:** 10.3390/ijms21249379

**Published:** 2020-12-09

**Authors:** Sha Yin, Qianwen Shi, Wenwei Shao, Chi Zhang, Yixiao Zhang, Xiaoyan Qiu, Jing Huang

**Affiliations:** 1 Department of Immunology, School of Basic Medical Sciences, Peking University, Beijing 100191, China; shayin@bjmu.edu.cn (S.Y.); 17695438074@163.com (Q.S.); sww6296613@126.com (W.S.); azuretimm@bjmu.edu.cn (C.Z.); zyx960525@163.com (Y.Z.); 2 NHC Key Laboratory of Medical Immunology, Peking University, Beijing 100191, China

**Keywords:** liver injury, hepatocyte, Igκ, apoptosis, ConA, mitochondria, NF-κB, NCTC1469

## Abstract

Immunoglobulin (Igκ) has been reported to be expressed in sorted liver epithelial cells of μMT mice, and the sequence characteristics of hepatocyte-derived Igκ were different from those of classical B-cell-derived Igκ. However, the physiological function of hepatocyte-derived Igκ is still unclear. The expression of Igκ was firstly identified in primary hepatocytes and normal liver cell line (NCTC1469), and hepatocyte-derived Igκ expression was elevated and displayed unique localization in hepatocytes of concanavalin A (ConA)-induced hepatitis model. Moreover, *Igκ* knockout mice were more sensitive to ConA-induced hepatitis and had higher serum aspartate aminotransferase (AST) levels, more severe histological injury and a greater number of terminal deoxynucleotide transferase-mediated deoxyuridine triphosphate nick end-labeling (TUNEL)-positive cells as compared with littermate controls. Furthermore, knockdown of *Igκ* in primary hepatocytes and NCTC1469 cells led to accelerated activation of the mitochondrial death pathway and caspase-3 cleavage in vitro, which might be related to inhibition of NF-κB signaling pathway and activation of JNK via the cytoskeleton dynamics. Taken together, these results indicate that hepatocyte-derived Igκ mediates cellular resistance to ConA-induced liver injury by inhibiting activation of caspase-3 and the mitochondrial death pathway, suggesting that Igκ plays an important role in hepatocyte survival and exerts a protective effect against ConA-induced liver injury in mice.

## 1. Introduction

According to traditional immunological theory, immunoglobulins (Igs) are generated with enormous diversity exclusively by B-lineage lymphocytes in response to various forms of antigen stimulation. The biological functions of classical B-cell-derived Igs have been extensively described. However, growing evidence indicates that non-B-cells can also produce Igs (non-B-Igs) with activities distinct from antibodies. Such cells include malignant tumor cells [1,2] and normal non-B-cells, including epithelial cells, skin epidermal cells, endothelial cells, neurons, germ cells, and even myeloid cells. These non-B-cells can express Igs with unique molecular characteristics, such as IgG, IgA, and IgM [3,4,5,6]. In particular, the sorted liver epithelial cells of B-cell-deficient μMT mice possess *Ig* heavy- and light-chain transcripts. In these mice, the *Ig* variable region displays distinct sequence characteristics, which differ from those of the classical B-cell-derived *Ig* variable region [7,8]. Moreover, liver epithelial cell-derived natural IgM contributes to innate immune responses. Besides these, the sequencing results from human or mouse hepatocytes in the Gene Expression Omnibus (GEO) Profile, which were provided by other research groups [9,10,11], were analyzed and revealed that these hepatocytes possess *Igκ* transcripts, suggesting that Igκ plays a role in the pathophysiological processes of the liver.

Igκ has been reported to be associated with clinical liver disease. For example, large-scale RNA-seq of human liver biopsy specimens revealed that the intrahepatic expression levels of *IgκC* in non-alcoholic steatohepatitis patients were significantly higher than those in healthy people [12]. Moreover, Ig light-chain deposition was detected by liver biopsy in patients with hepatomegaly [13], and Igκ chain deposition disease of the liver is related to liver failure and rapid fatal outcomes [14]. Generally, Ig light chains are linked to Ig heavy chains by disulfide bonds to form tetrameric *Ig*. However, more free Ig light chains (FLCs) exist as monomers (molecular weight 22–27 kDa) and as covalently or non-covalently bound dimers (44–55 kDa) or polymers in blood, urine, and cerebrospinal fluid. FLCs, which have been the subject of great scientific interest and proposed as a biomarker for a number of autoimmune and chronic inflammatory conditions, might lead to irreversible systemic amyloidosis and death.

Moreover, clinical data and animal models suggest that hepatocyte death is the key trigger for liver disease progression, which manifests as the subsequent development of inflammation, fibrosis, cirrhosis, and hepatocellular carcinoma [15]. In certain pathophysiological settings, such as concanavalin A (ConA)-induced liver injury, activation of the mitochondrial death pathway triggers the release of cytochrome c and other factors, which activate downstream effector caspases to induce hepatocyte apoptosis [16,17]. A critical hepatocyte event in this cell death cascade is the inhibition of nuclear factor-κB (NF-κB) signaling [18]. In an NF-κB essential modulator (NEMO)-deficient model of chronic liver disease, intrahepatic *Igκ v9-120* levels were significantly elevated in NEMO-knockout mice and in NEMO/TRAIL double knockout mice [19]. More importantly, our previous work showed that knockout of *Igκ* in normal epithelial cells promotes renal epithelial cell apoptosis [20,21]. Collectively, these data suggest a possible close relationship between Igκ and hepatocyte death-induced liver disease.

In this study, we first confirmed the expression of Igκ in mouse primary hepatocytes and a normal hepatocyte cell line. To further investigate the role of hepatocyte-derived Igκ, we demonstrated that the absence of *Igκ* accentuated liver injury upon ConA challenge and found that Igκ is essential for hepatocyte survival. Furthermore, knockout of *Igκ* led to accelerated activation of the mitochondrial death pathway and caspase-3 cleavage in vitro. These findings demonstrate a critical role of hepatocyte-derived Igκ in hepatocyte survival and indicate that Igκ has a protective effect against ConA-induced liver injury in mice.

## 2. Results

### 2.1. Igκ Expression in Primary Hepatocytes in μMT Mice and Normal Hepatocyte Cell Line

Previous studies have demonstrated that various classes of Ig are produced by liver epithelial cells in B-cell-deficient μMT mice [7]. We investigated whether the *Igκ* gene transcript was expressed in human or mouse hepatocytes through integrative analysis of Gene Expression Omnibus (GEO) profile data. As shown in Table 1, *Igκ* gene transcripts with different *VκJκ* rearrangements were found in mouse and human hepatocytes.

**Figure 1:**
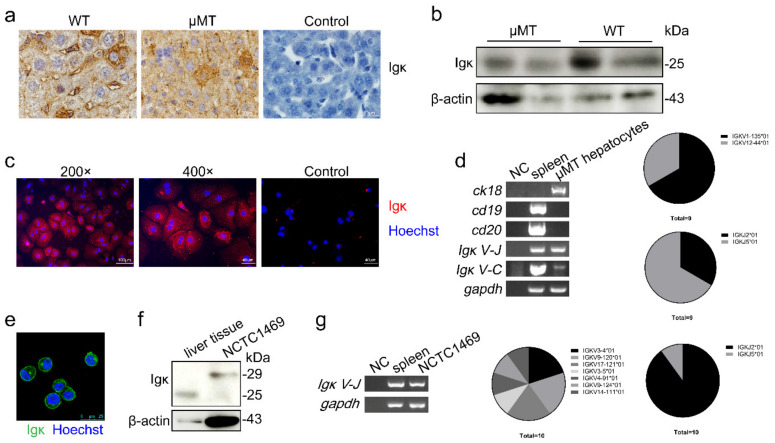
Expression of Igκ in mouse primary hepatocytes and a normal hepatocyte cell line. (**a**) Immunohistochemistry and (**b**) Western blot analysis of Igκ in wild type (WT) and μMT mouse liver tissue. Control indicates isotype control. (**c**) The magnification and immunofluorescence analysis of Igκ in primary hepatocytes in μMT mice. Rabbit IgG as isotype control. (**d**) Reverse-transcription PCR analysis of *ck18*, *cd19*, *cd20*, *Igκ V-J*, *Igκ J-C*, and *gapdh in* primary hepatocytes in μMT mice. WT spleen cells were used as the positive control, and NC was used as the negative control. The frequency of *Vκ* and *Jκ* derived from primary hepatocytes in μMT mice is displayed on the right. (**e**) Immunofluorescence analysis of Igκ in the NCTC1469 cell line. (**f**) Western blot analysis of Igκ in μMT liver tissue and the NCTC1469 cell line. (**g**) Reverse-transcription PCR analysis and *VκJκ* rearrangement patterns of *Igκ* in the NCTC1469 cell line.

To further confirm the expression pattern of Igκ in hepatocytes, we first performed immunohistochemistry (IHC) using anti-mouse Igκ antibody to determine the expression level of Igκ in hepatocytes of wild type (WT) (Balb/c background) and μMT mice. Igκ was mainly localized in the cytoplasm of the liver tissue cells. Significant Igκ staining was observed in a small population of hepatocytes (Figure 1a). Igκ was also detected in liver tissue perfused with phosphate-buffered saline (PBS) through the hepatic portal vein on Western blot analysis. The level of liver-derived Igκ in μMT mice was less than that in WT mice (Figure 1b). Furthermore, we sought to determine the Igκ staining pattern in single mouse primary hepatocytes isolated from μMT mice using a two-step collagenase perfusion method. Igκ staining revealed a cytoplasmic filamentous network and pointed shape around the nucleus (Figure 1c). Interestingly, Igκ was partially co-localized with cytokeratin 18 (CK18) in primary hepatocytes (Figure S1).

Next, we used RT-PCR to determine the expression of Igκ in primary hepatocytes, in which the epithelial cell marker *cytokeratin 18 (ck18)* but not the B-cell markers *cd19* and *cd20* was detected. Importantly, rearranged transcripts of *Igκ* were clearly detected in the primary hepatocytes (Figure 1d), and the *Igκ* variable region displayed distinct sequence characteristics, such as a restricted *VκJκ* recombination pattern, in agreement with our previous findings (i.e., IGKV12–44*01 and IGKJ2*01). In addition, we found that Igκ was localized on the cell membranes and cytoplasm of normal hepatocyte cell lines (NCTC1469) (Figure 1e). Igκ expression was also confirmed in NCTC1469 at the protein and mRNA levels by Western blotting and RT-PCR (Figure 1f,g). There were two forms (25 kDa and 29 kDa) of the Igκ protein in the NCTC1469 cell line, and these differed from those found in liver tissue. This resulted from the N-glycosylation of Igκ (unpublished data).

### 2.2. Hepatocyte-Derived Igκ Was Elevated and Displayed Unique Localization in ConA-Induced Liver Injury in μMT Mice

**Figure 2:**
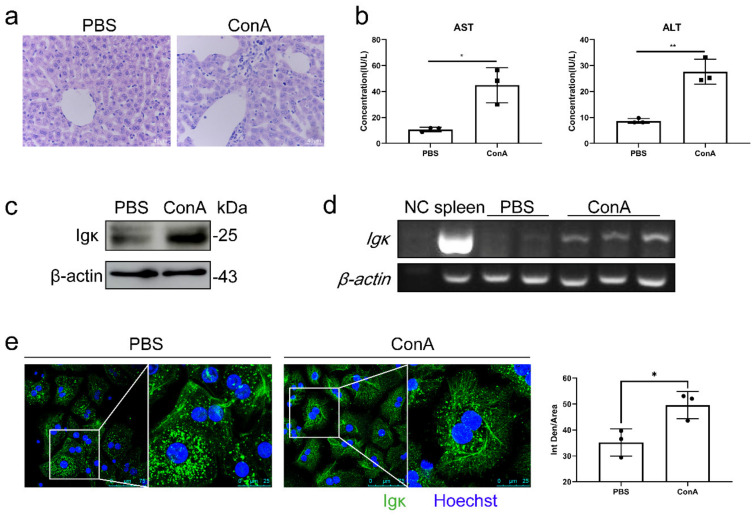
Upregulation and subcellular localization of Igκ in concanavalin A (ConA)-induced liver injury in μMT mice. The μMT mice were treated with ConA or PBS and sacrificed 24 h later. (**a**) Liver sections of μMT mice were stained with hematoxylin and eosin and representative images are shown. (**b**) Levels of serum alanine aminotransferase and aspartate aminotransferase. * *p* < 0.05; ** *p* < 0.01. (**c**) Western blot and (**d**) Reverse-transcription PCR analysis of *Igκ* and *β-actin* in primary hepatocytes. (**e**) Immunofluorescence staining of Igκ in primary hepatocytes. Quantification of fluorescence intensity was performed using ImageJ version 1.52. Data are shown as mean ± SEM (3 different fields). Statistical analysis was performed by unpaired two-tailed Student’s *t*-test. * *p* < 0.05; ** *p* < 0.01.

Based on the relationship between Igκ expression and clinical liver disease, we further detected Igκ expression during liver injury. We administered ConA or PBS to μMT mice, and liver injury was measured by liver histology. The serum levels of AST and alanine aminotransferase (ALT) were obviously affected in the ConA-treated mice. The histological evaluation indicated significant hepatocyte apoptosis, loss of cellular integrity, and massive immune cell infiltration into the parenchyma in the ConA-treated μMT mouse livers (Figure 2a). The serum ALT and AST levels were significantly elevated in the ConA-treated μMT mice (Figure 2b). Moreover, Igκ expression in primary hepatocytes was elevated at the protein and mRNA levels during ConA-induced liver injury (Figure 2c,d). Surprisingly, Igκ showed an increased cytoplasmic filamentous network but remained abundant in a point shape around the nucleus in the ConA-treated hepatocytes (Figure 2e). These observations indicate that hepatocyte-derived Igκ might play an important role in ConA-induced liver injury.

### 2.3. Target Disruption of Igκ Accentuated ConA-Induced Liver Injury In Vivo

**Figure 3:**
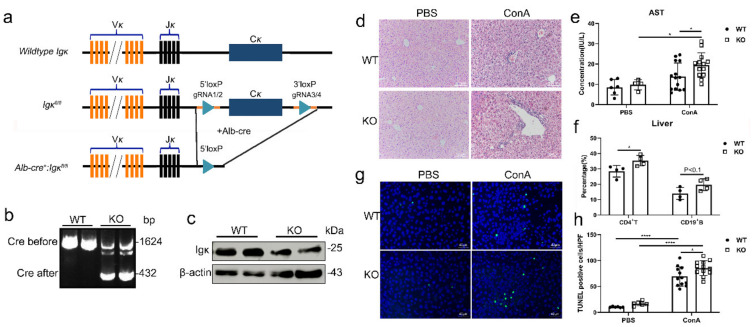
Hepatocyte-derived Igκ inhibited ConA-induced liver injury in vivo. The wild type (WT) and knockout (KO) mice were treated with ConA or PBS and sacrificed 24 h later. (**a**) Scheme for generation of hepatocyte-specific Igκ knockout mice. LoxP sites were inserted to flanking exon in *Igκ* gene constant region. Mice homozygous for loxP insertion (fl/fl) were crossed with mice expressing Cre recombinase under control of the Alb-cre promoter. (**b**) PCR analysis of *Igκ* transcript in liver tissue of wild type (WT) and knockout (KO) mice. (**c**) Western blot analysis of Igκ protein in liver tissue of WT and KO mice. (**d**) Liver sections of WT and KO mice were stained with hematoxylin and eosin and representative images are shown. (**e**) Levels of serum AST were measured. (**f**) Percentage of liver infiltrated lymphocytes was analyzed in WT and KO mice stimulated by ConA or phosphate-buffered saline (PBS). (**g**) Transferase-mediated deoxyuridine triphosphate nick end-labeling (TUNEL) analysis of primary hepatocytes in WT and KO mice. (**h**) The number of TUNEL-positive cells per high power field was calculated. The results presented were from three independent experiments, and each error bar represents the standard deviation. Statistical analysis was performed by one-way analysis of ANOVA. * *p* < 0.05; **** *p* < 0.0001.

To further investigate the role of hepatocyte-derived Igκ in vivo in ConA-induced liver injury, we generated hepatocyte-specific *Igκ* knockout (*Alb-cre^+^:Igκ^fl/fl^*, KO) mice. Genomic analysis of the *Igκ* KO mice confirmed that treatment with Alb-cre led to knockout of *Igκ* (Figure 3a). Western blot analysis revealed a significant reduction in Igκ protein at 25 kDa in livers of *Igκ* KO mice compared to wild-type (*Alb-cre^−^:Igκ^fl/fl^*, WT) control littermates, indicating that our mouse model resulted in Igκ protein deficiency in the liver (Figure 3b). The hepatocyte-specific *Igκ* KO mice appeared to have a normal life span and were fertile (both females and males) when compared with the controls. Further, they did not show any behavioral abnormalities.

Next, the WT and KO mice were treated with PBS or ConA, and the deletion of *Igκ* led to more severe liver injury with ConA treatment. This manifested in changes in liver histology and serum AST levels when compared with the control group (Figure 3c,d). Moreover, the hepatocyte-specific *Igκ* knockout promoted an increase in the proportion of CD4^+^ T cells and CD19^+^ B cells in the liver and spleen infiltrating lymphocytes in the ConA-induced liver injury model, and upregulated the expression of serum inflammatory factors, especially IL-6 (Figure 3e, Figure S2).

Because ConA activates T-cells, which in turn produce a number of cytokines such as TNFα and IL-6, which induce liver injury related to hepatocyte apoptosis, we next examined whether deletion of Igκ promoted liver injury by inhibiting hepatocyte survival. As shown in Figure 3f,g, there was more hepatocyte apoptosis in the liver tissue of the KO mice than the WT mice on terminal deoxynucleotidyl transferase dUTP nick end labeling staining. Taken together, these results indicated that hepatocyte-derived Igκ might protect hepatocytes from ConA-induced liver injury through inhibiting hepatocyte apoptosis.

### 2.4. Knockout of Igκ Inhibited Hepatocyte Survival and Promoted Hepatocyte Apoptosis

**Figure 4:**
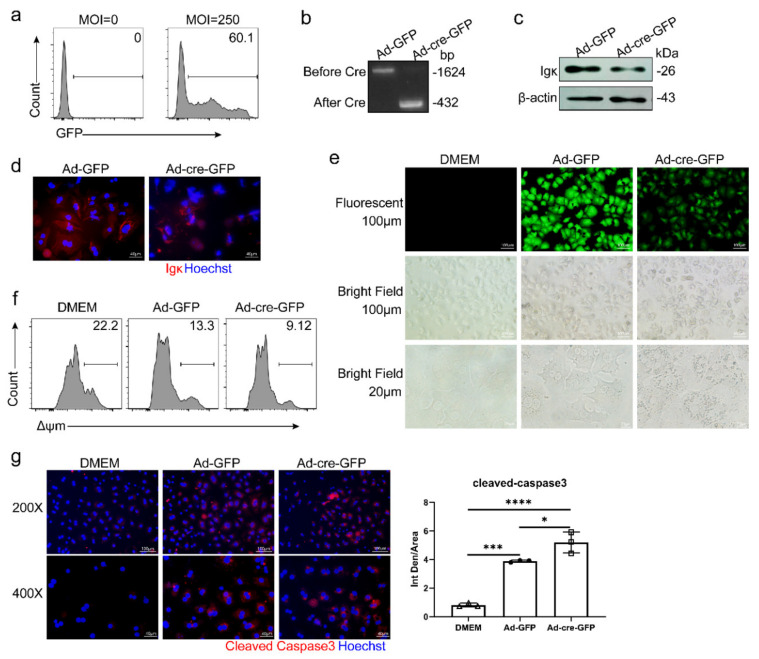
Knockout of *Igκ* promoted a potent mitochondrial apoptotic response and caspase-3 activation in hepatocytes in vitro. Primary hepatocytes isolated from *Igκ^fl/fl^* mice were infected with Ad-cre-GFP or Ad-GFP for 24 h. (**a**) Flow cytometry analysis of infection efficiency of primary hepatocytes infected with Ad-GFP (multiplicity of infection = 250). Identification of *Igκ* knockout efficiency was accomplished using a genomic PCR assay (primers for both sides of the flox region were designed). The PCR product before Cre was 1624 bp, and the PCR product after Cre was 432 bp, implying that *Igκ* was knocked out in hepatocytes. (**b**) Western blot analysis (**c**) immunofluorescence analysis (**d**,**e**) Fluorescent and bright-field images of primary hepatocytes infected with Ad-cre-GFP or Ad-GFP for 24 h. (**f**) Analysis of mitochondrial membrane potential in primary hepatocytes by fluorescence-activated cell sorting. (**g**) Representative immunofluorescence staining of cleaved caspase-3. DAPI (blue) was used for nuclear staining. Scale bars, 100 μm (left) and 40 μm (right). Quantification of fluorescence intensity was performed using ImageJ version 1.52. Data are shown as mean ± SEM (average of 4 different fields from 3 independent experiments). Statistical analysis was performed by unpaired two-tailed Student’s *t*-test. * *p* < 0.05; *** *p* < 0.001; **** *p* < 0.0001.

To explore the physiological and pathological significance of Igκ in primary hepatocytes, we isolated primary hepatocytes from *Igκ^fl/fl^* mice in vitro and infected cells with adenovirus harboring either cre recombinase fusion with GFP (Ad-cre-GFP) or GFP alone (Ad-GFP) as controls. The infection efficiency of Ad-GFP was calculated; the efficiency reached as high as 60% (multiplicity of infection = 250) (Figure 4a). The PCR assay, Western blot analysis, and immunofluorescence staining confirmed that Ad-cre-GFP successfully knocked out *Igκ* in primary hepatocytes (Figure 4b–d). It is worth noting that knockout of primary hepatocyte-derived *Igκ* in vitro led to less Igκ cytoplasmic filamentous network staining (Figure 4d), which was also observed in primary hepatocytes treated with ConA and lipopolysaccharide (LPS), suggesting that hepatocyte-derived Igκ might be related to the cytoskeleton (Figure S3).

More interestingly, Ad-cre-GFP-infected cells had more intracellular vacuoles and were in worse condition than Ad-GFP-infected cells after 12 h of infection (Figure 4e). Previous studies have shown that the induction of the intrinsic pathway of apoptosis in hepatocytes requires mitochondrial participation and caspase-3 activation. Next, the Δψm of primary hepatocytes was detected using Mito-Tracker Red CMXRos. Although adenovirus infection reduced the Δψm of primary hepatocytes compared with the control group, knockout of *Igκ* decreased Δψm even more (Figure 4f). Consistently, knockout of *Igκ* promoted caspase-3 activation in primary hepatocytes (Figure 4g). These observations indicated that knockout of *Igκ* promoted a potent mitochondrial apoptotic response and caspase-3 activation in primary hepatocytes.

### 2.5. Knockout of Igκ Promoted Mitochondria-Mediated Apoptosis in NCTC1469

**Figure 5:**
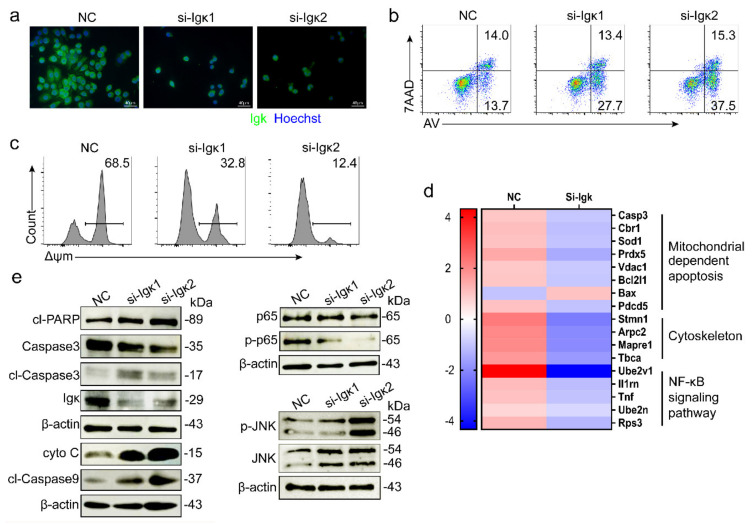
Knockout of *Igκ* induced mitochondria-mediated apoptosis in the NCTC1469 cell line. NCTC1469 cells were transfected with si-*Igκ* or control si-RNA (NC) for 24 h before analysis. (**a**) Bright-field images and immunofluorescence staining of Igκ. (**b**) *Igκ* knockout promoted NCTC1469 cell apoptosis on Annexin V and 7-AAD analysis. (**c**) *Igκ* knockout in NCTC1469 cells reduced mitochondrial membrane potential. (**d**) Protein mass spectrometry analysis of NCTC1469 cells transfected with si-*Igκ* and NC. (**e**) Western blot analysis of cleaved poly (ADP-ribose) polymerase, caspase-3, cleaved caspase-3, Igκ, cleaved caspase-9, cytochrome c, p65, p-p65, p-JNK, JNK and β-actin after *Igκ* knockout in NCTC1469 cells.

To further explore the mechanism of hepatocyte-derived Igκ’s role in hepatocyte survival, we used a normal hepatocyte cell line, NCTC1469, to detect Igκ function in vitro. We found that knockout of *Igκ* in NCTC1469 led to a worse cell state and increased cell apoptosis (Figure 5a,b). Similar to the results of *Igκ* knockout in primary hepatocytes, *Igκ* knockout significantly decreased Δψm in NCTC1469 (Figure 5c). Furthermore, we performed protein mass spectrometry and found that knockout of *Igκ* might induce mitochondria-dependent apoptosis and changes to the cytoskeleton and NF-κB signaling pathway (Figure 5d). As shown in Figure 5e, increased levels of cytochrome c in the cytosolic fractions of NCTC1469 cells were detected after *Igκ* knockout. Mitochondrial cytochrome c release initiates cleavage of downstream effector caspases that mediate cell apoptosis. In parallel with increased mitochondrial cytochrome c release, cleavage and activation of effector caspases -3 and -9 and cleavage of the caspase substrate poly (ADP-ribose) polymerase (PARP) were detected in NCTC1469 cells transfected with si-*Igκ*. Moreover, *Igκ* knockout inhibited the NF-κB pathway but increased the phosphorylation levels of JNK. As a result, knockout of *Igκ* in NCTC1469 cells induced mitochondria-mediated apoptosis, suggesting *Igκ* knockout mice might be slightly more sensitive than controls to ConA-induced liver injury.

## 3. Discussion

To date, Ig has been considered a product of B-cells that acts as an antibody. However, growing evidence shows that malignant tumor non-B cells can produce Ig and that this process is involved in tumor growth and metastasis [2,22]. Furthermore, normal non-B-cells, including liver epithelial cells, produce multiple classes of *Igs* with distinctive characteristics in the variable region [7]. Although considerable progress concerning the understanding of the expression and characteristics of hepatocyte-derived Ig has been made, its functions are still incompletely understood. Based on bioinformatics analysis and our previous studies, Igκ is highly expressed in hepatocytes (Table 1) and might be closely related to liver disease [12,19]. The aim of the current study was to evaluate the expression and function of Igκ in hepatocytes and liver injury in vivo. Initial attempts to determine the expression of Igκ in primary hepatocytes and a normal hepatocyte cell line NCTC1469 were made. The *Igκ* variable region exhibited the same restricted *VκJκ* recombination pattern as observed previously, further confirming that Igκ can be produced by hepatocytes but is not limited to B-cells as previously reported.

Growing evidence indicates that Igκ deposition is significantly linked to the progression and severity of clinical liver disease, such as liver injury. In this study, we used a ConA-induced liver injury mouse model to show that Igκ expression was significantly enhanced in the hepatocytes of ConA-induced mice. Interestingly, increasing Igκ levels manifested in cytoplasmic filamentous network but remained dotted distribution around the nucleus in ConA-treated hepatocytes. Our previous results showed that FLCs were significantly increased in the colon tissue of dextran sulfate sodium salt (DSS)-induced colitis mice and that F991, an FLC inhibitor, significantly suppressed the progression of DSS-induced colitis [23]. The above data suggest that Igκ deposition in ConA-treated hepatocytes might be associated with inflammation and liver injury.

To further address the role of Igκ in liver injury, we deleted the constant region of the *Igκ* gene in hepatocytes using the cre–loxP system. Albumin-driven expression of cre-recombinase resulted in the loss of *Igκ* mRNA and protein in hepatocytes. As expected, hepatocyte-specific deletion of *Igκ* resulted in increased liver injury upon administration of ConA, including histologic liver damage and increasing serum ALT and AST levels. The ConA-induced liver injury model was originally thought to represent a T-cell-driven liver injury model [24]. ConA induced acute liver damage within 8 h in a dose range of 10–25 mg/kg, and bound to non-parenchymal cells in the liver, such as endothelial cells, Kupffer cells, and CD4^+^ T-cells, resulting in the secretion of pro-inflammatory cytokines such as MCP-1 and IL-6 and finally leading to inflammation and hepatocyte damage characterized by apoptotic cell death [17,25]. In addition, the hepatocyte death induced by ConA was accompanied by the release of the aminotransferases ALT and AST from the cytoplasm of hepatocytes into the blood. These findings further indicate that hepatocyte-derived *Igκ* deficiency accentuated ConA-induced liver injury.

We next identified the mechanisms of *Igκ*-mediated liver injury in a ConA-induced mouse model. Liver cell injury in *Igκ* KO mice with ConA treatment was accompanied by apoptotic cell death and activation of caspases. Moreover, knockout of *Igκ* in primary hepatocytes or the NCTC1469 cell line in vitro promoted cell apoptosis through the mitochondrial pathway. It has been reported that ConA induces cell apoptosis in vitro in a caspase-dependent manner as well as via a mitochondrial apoptotic pathway, which appears to reduce mitochondrial membrane potential, release cytochrome c, and activate caspase-9 and -3 [16,26]. This finding can be explained by the amplification of the cell death signal in hepatocytes following the liberation of pro-apoptotic factors from mitochondria. We observed a pronounced decrease in mitochondria-dependent apoptosis-related proteins after *Igκ* knockout in NCTC1469 cells, indicating cleavage and activation of this pro-apoptotic protein.

The nuclear transcription factor NF-κB is a critical regulator of genes involved in immune response, inflammation, and cell apoptosis [27]. NF-κB activation is also implicated in liver susceptibility to apoptosis-inducing stimuli, such as ConA and TNF-α [28]. In the present study, we also found that knockout of *Igκ* in the NCTC1469 cell line significantly inhibited p-p65 activity and increased phosphorylation of JNK, finally promoting mitochondria-dependent apoptosis. Initial experiments found that NF-κB inactivation led to a prolonged activation of JNK and increased activating protein-1 (AP-1) transcriptional activity in response to TNF-α treatment, which initiates mitochondria-dependent cell apoptosis [29,30]. Other study has reported that increased proapoptotic effect of ConA correlates with its ability to elicit persistent JNK activation in hepatocytes after knockout of IKKβ, which is critical for IκB degradation and activation of NF-κB in response to proinflammatory stimuli. Among hepatocytes, hepatocytes deficient for IKKβ show residual NF-κB activity, whereas primary RelA/p65-deficient hepatocytes show no NF-κB activation. This means that JNK is more continuously activated after TNF-α treatment, leading to rapid apoptosis [31,32]. These results suggest that Igκ-mediated liver injury might result from mitochondria-dependent apoptosis via inactivation of NF-κB and activation of JNK.

Interestingly, Igκ staining in hepatocytes also showd a cytoskeleton-like cytoplasmic filamentous network structure, which was partially co-localized with CK18 staining. More importantly, after knockout of *Igκ* in hepatocytes, ConA stimulation reduced Igκ cytoplasmic filamentous network staining, whereas perinuclear dot deposits of Igκ remained in vitro and in vivo. The changes in Igκ localization after LPS stimulation in vitro were similar. CK8 and CK18 are the only keratins found in adult hepatocytes [33] and serve several important mechanical and non-mechanical cellular functions, such as protection from apoptosis [34]. During apoptosis, CK18 undergoes caspase-mediated digestion, which leads to dramatic disassembly of keratin filaments. Caspase digestion-resistant CK18 helps to maintain keratin filament organization and delays apoptosis, resulting in protection from liver injury [35]. The deletion or mutation of *CK8* or *CK18* affects the shape and function of the mitochondria in liver cells, leading to liver injury and apoptosis [34]. In addition, the results of protein mass spectrometry in NCTC1469 cells indicated that the knockout of *Igκ* affected cytoskeleton-related proteins such as STMN1, which is involved in the regulation of the microtubule filament system by destabilizing microtubules, induced murine hepatocyte proliferation, and increased liver mass [36]. These findings suggest that Igκ may be a novel cytoskeleton binding partner to regulate hepatocyte apoptosis.

The results of this study raised a few unanswered questions. First, we detected free Igκ and intact Ig consisting of Igκ and Ig heavy chains in hepatocytes. The size of the monomer Igκ chains was 25 kDa in the liver tissue of ConA-induced mice, whereas the size of the Igκ chains in the NCTC1469 cell line was 29 kDa. These larger chains were identified as N-glycosylated. However, it remained unclear what form of Igκ in hepatocytes plays a protective role against ConA-induced liver injury. Second, although Igλ redundancy in hepatocyte-specific *Igκ* KO mice was not detected by Western blot analysis, the mechanism needs to be further investigated to identify whether Igλ plays a role in ConA-induced liver injury.

In summary, the results of this study support the hypothesis that hepatocyte-derived Igκ plays a protective role in ConA-induced liver injury. The loss of Igκ resulted in liver damage involving the activation of caspases and NF-κB to promote hepatocyte apoptosis. These findings might provide new strategies and potential targets for the modulation of acute liver injury in the clinical setting.

## 4. Materials and Methods

### 4.1. Animals

The μMT mice (Balb/c background) were a gift from Professor Zhihai Qin (Institute of Biophysics, Chinese Academy of Sciences). For the generation of *Igκ^fl/fl^* heterozygous mice, four guide sequences (upstream: 5′-tccatacagtaggtttagct-3′ and 5′-agcctctgtatggcttcctt-3′; downstream: 5′-aggttcacgagtactattca-3′ and 5′-ttatttctctgggccatggt-3′) were targeted to the flanking exon in the *Igκ* gene constant region and cloned into the pT7-Guide Vector (mMESSAGE mMACHINE® T7 Ultra Kit, AM1345, Life Technologies, USA). The guide RNAs were in vitro transcribed from the pT7-Guide Vector using the MEGAshortscript T7 kit (AM1354; Life Technologies, Waltham, MA, USA), and products were subsequently purified using the MEGAclear kit (AM1908; Life Technologies, Waltham, MA, USA). Using the pT7-Cas9-Nuclease vector, the Cas9 messenger RNA (mRNA) was in vitro transcribed using the mMESSAGE mMACHINE T7 ULTRA kit (AMB13455; Life Technologies, Waltham, MA, USA) and purified using the MEGAclear kit (AM1908; Life Technologies, Waltham, MA, USA). C57BL/6 background female mice were superovulated and mated with C57BL/6 background male mice, and one-cell stage embryos were collected for microinjection. Clustered regularly interspaced short palindromic repeats (CRISPR) reagents were microinjected at the following concentrations: Cas9 mRNA (100 ng/μL), single guide RNA (sgRNA) (50 ng/μL), and DNA oligo (50 ng/µL). Injected zygotes were transferred into pseudopregnant females, and resulting progeny were initially screened for potential recombination events via PCR. PCR primers 5′-ctggcagttgcttaagatca-3′ and 5′- acatttaggttgcctttgct-3′ were used to screen for loxP insertion. Next the Igκfl/fl mice and Alb-cre transgenic mice were mated to generate Al*b-cre^−^:Igκ^fl/fl^* (wild-type, WT) mice and *Alb-cre^+^:Igκ^fl/fl^* (knockout, KO) mice. All experimental procedures were approved by the Peking University Laboratory Animal Research Committee on August 22, 2017 (LA2017224). The conditions of animal housing and all experimental procedures adhered to the institutional guidelines provided by the Institutional Animal Care and Use Committee of China.

### 4.2. ConA-Induced Liver Injury

Six-to-eight-week-old male μMT, WT, and KO mice were randomly divided into two groups. The control group mice were injected through the tail vein (intravenously, i.v.) with phosphate-buffered saline (PBS), and the ConA-treated group mice were injected (i.v.) with 15 mg/kg of ConA (Sigma, St. Louis, MO, USA) to induce acute liver injury.

### 4.3. Histology and Immunohistochemistry

Liver pathology was examined 8 h after ConA injection. The mice were anesthetized, and PBS was perfused through the heart to remove the blood. Then, the liver tissues were fixed in 10% buffered formalin and embedded in paraffin. The tissue sections were cut and stained with hematoxylin and eosin to observe the level of inflammation and tissue damage using light microscopy.

The liver tissues were subjected to immunohistochemistry (IHC). After normal procedures, primary rabbit anti-mouse Igκ antibody (Proteintech, Rosemont, IL, USA)) was added, followed by incubation at 4 °C overnight. The samples were washed with PBS three times and incubated with horseradish peroxidase (HRP)-conjugated anti-rabbit IgG (ZSGB-BIO) for 30 min at room temperature. All samples were developed with diaminobenzidine (DAB) (DakoCytomation, Carpinteria, CA, USA) after rinsing with PBS. Samples without primary antibodies added were used as negative controls.

### 4.4. Serum Biochemistry

Blood was collected 8 h after ConA injection, and the serum levels of alanine aminotransferase (ALT) and aspartate aminotransferase (AST) were measured using standard enzymatic procedures according to the manufacturer’s instructions (Nanjing Jiancheng Bioengineering Institute, Nanjing, China).

### 4.5. Isolation of Primary Mouse Hepatocytes

Primary hepatocytes were isolated from the mouse livers using the two-step ethylenediaminetetraacetic acid (EDTA) and collagenase perfusion method. After each mouse was anesthetized, the liver was perfused with Hank’s Balanced Salt Solution (HBSS) through the hepatic portal vein to remove the blood (flow rate = 7–9 mL/min for 5 min). The liver was then perfused with 100 CDU/mL collagenase IV (Solarbio, Beijing, China) in Dulbecco’s Modified Eagle’s Medium (DMEM) until the liver became soft. Next, the liver was removed and gently minced, and the released cells were dispersed in DMEM containing 10% fetal bovine serum (FBS) and 1% penicillin/streptomycin. The solution containing the mixed cells and debris was passed through a 100 µm cell strainer. Subsequently, the filtrate was centrifuged at 50× *g* for 3 min at 4 °C. The isolated cells were washed three times with DMEM and then seeded in collagen-coated plates. The cells were maintained in DMEM containing a high glucose concentration (4.5 g/L) supplemented with 10% FBS, 100 U/mL penicillin/streptomycin, and 0.1 μM dexamethasone for 4 h at 37 °C in a humidified atmosphere (5% CO_2_).

### 4.6. Adenovirus Infection of Primary Mouse Hepatocytes

After the isolation of primary hepatocytes and adherent culture for 4 h, the mixture was washed once with DMEM. Then, the primary hepatocytes were infected with adenovirus (HanBio, Shanghai, China) at a multiplicity of infection (MOI) of 250 for 16 h and stimulated with ConA (50 μg/μL) or PBS for 12 h after infection. The *Igκ* knockout hepatocytes were infected with Ad-cre-GFP adenovirus, and the control group was infected with Ad-GFP.

### 4.7. NCTC1469 Cell Culture and Transient Transfection

A mouse normal hepatocyte cell line (NCTC1469) was obtained from the Cell Resource Center (Institute of Basic Medical Sciences, Chinese Academy of Medical Sciences & Peking Union Medical College). The cells were cultured in DMEM supplemented with 10% horse serum (Beyotime Biotechnology, Nanjing, Jiangsu, China) and 100 U/mL penicillin/streptomycin at 37 °C in a humidified atmosphere (5% CO_2_).

Before transfection, 2.5 × 10^5^ cells per well were seeded in a 6-well plate with 2 mL DMEM culture medium containing serum and antibiotics. Meanwhile, negative control si-RNA and two specific si-RNA against *Igκ* were transfected into the NCTC1469 cells. The transfected cells were incubated under normal growth conditions for another 12 h.

### 4.8. Immunofluorescence Analysis

Primary hepatocytes and NCTC1469 cells were fixed with acetone and blocked with 5% FBS at room temperature for 30 min. Rabbit anti-mouse Igκ (Proteintech, Rosemont, IL, USA) was added at 4 °C overnight. After washing with PBS, FITC-conjugated anti-rabbit (ZSGB-BIO, Beijing, China) and tetramethylrhodamine–isothiocyanate (TRITC)-conjugated anti-rabbit IgG (ZSGB-BIO, Beijing, China) were added at room temperature for 1 h. Sections were visualized and imaged with a fluorescence microscope (BZ-X700; Keyence, Osaka, Japan) or a confocal laser-scanning microscope (LSM 800; Carl Zeiss, Oberkochen, Germany). Quantification of fluorescence intensity for Igκ or cleaved caspase-3 was performed using ImageJ version 1.52.

### 4.9. Mitochondrial Membrane Potential and Apoptosis Analysis by Flow Cytometry

To detect changes in the mitochondrial membrane potential (ΔΨm), primary hepatocytes or NCTC1469 cells treated with the different reagents were incubated with 100 nM Mito-Tracker Red CMXRos diluted in DMEM at 37 °C in the dark for 30 min. Then, the fluorescence intensity was determined by flow cytometry to measure ΔΨm.

Apoptosis analysis was performed using an Annexin V-FITC/7AAD apoptosis detection kit (BD Biosciences, San Diego, CA, USA). The cells were collected, washed twice with ice-cold PBS, and stained with Annexin V-FITC for 30 min and then with 7AAD for 5 min. The distributions of viable, early-apoptotic, late-apoptotic, and necrotic cells were analyzed via flow cytometry. The data were analyzed using FlowJo software according to the manufacturer’s instructions.

### 4.10. Reverse-Transcription PCR and Sequencing Analysis of the Igκ Gene Transcripts

Total RNA was extracted from primary hepatocytes and NCTC1469 cells using Trizol Reagent (Invitrogen, Waltham, MA, USA). Reverse transcription (RT) was performed using a First Strand cDNA Synthesis Kit (Thermo Fisher Scientific, Waltham, MA, USA) according to the manufacturer’s protocol. After the cDNA was obtained, specific primers were used to amplify the *Igκ* variable and constant regions.

The PCR products were cloned into a pGEM-T Easy Vector (Promega, Madison, Wisconsin, USA). These clones were analyzed using the Sanger method with an ABI 3730XL Genetic Analyzer (Applied Biosystems, Waltham, MA, USA). The sequences of *VκJκ* were compared to those in the BLAST and Immunogenetics databases to identify the best matches for germline gene segments and VJ junctions.

### 4.11. Western Blot Analysis

The cells were directly lysed in culture dishes with radioimmunoprecipitation assay (RIPA) buffer supplemented with a proteinase and phosphatase inhibitor cocktail mixture. The cell lysates (30 µg) were separated using 12% sodium dodecyl sulfate (SDS)-polyacrylamide gel electrophoresis and then transferred to polyvinylidene fluoride membranes. The blots were then washed with tris-buffered solution containing Tween-20 (TBST: 10 mM Tris-HCl, 150 mM NaCl, and 0.1% Tween-20), blocked with 5% non-fat milk in TBST for 1 h at room temperature, and incubated for 12 h at 4 °C with the appropriate primary antibodies against Igκ, cleaved poly (ADP-ribose) polymerase (PARP), cleaved caspase-3, cleaved caspase-9, cytochrome C, phosphorylated p65 (p-p65), p65, phosphorylated JNK (p-JNK), JNK and β-actin at a 1:1000 dilution, which were purchased from Cell Signaling Technology (CST, Boston, MA, USA). The membranes were then washed with TBST and incubated with HRP-conjugated goat anti-rabbit or goat anti-mouse IgG antibodies (1:10,000 dilution) for 1 h at room temperature. The bands were visualized using an enhanced chemiluminescence detection system (Thermo Scientific, Waltham, MA, USA) according to the manufacturer’s protocols.

### 4.12. Statistical Analysis

The data were represented as the mean ± standard deviation of at least three independent experiments. Statistical analyses were performed using GraphPad Prism version 7.0 software (GraphPad Software Inc., San Diego, CA, USA). Differences between groups were assessed using a two-tailed unpaired Student’s t-test or analysis of variance for the comparison of two or multiple groups, respectively. The differences were considered statistically significant if the *p*-value was less than 0.05.

## Figures and Tables

**Table 1:** *Igκ* expression in human or mouse hepatocytes from Gene Expression Omnibus (GEO) Profiles.

Dataset *	Title	Organism	*Igκ*
GDS5673	Glucagon effect on hepatocytes deficient in lysine acetyltransferase 2B or WD repeat-containing protein 5	Mus musculus	*Igκv4–9* *Igκv10–96*
GDS3148	Hepatocyte growth factor effect on Met receptor-knockout primary hepatocytes: time course	Mus musculus	*Igκv1–117*
GDS1648	Hypoxia effect on HIF-1 alpha null hepatocytes	Mus musculus	*Igκv6–23* *Igκv16–104*
GDS4327	Human hepatocytes from xenogeneic host livers	Mus musculus Homo sapiens	*Igκ*

* Dataset is from GEO Database of NCBI.

## Supplementary Materials

The following are available online at https://www.mdpi.com/1422-0067/21/24/9379/s1, Figure S1: Immunofluorescence staining of CK18 and Igκ in primary hepatocytes, Figure S2: Hepatocyte-derived Igκ inhibited ConA-induced liver injury *in vivo*. WT and KO mice were stimulated by PBS or ConA for 24h and sacrificed 24 h later, Figure S3. Knockout of *Igκ* promoted hepatocytes apoptosis after ConA or LPS treatment *in vitro*.
