# Supplementary material for: Hepatocyte-Derived Igκ Exerts a Protective Effect against ConA-Induced Acute Liver Injury
Source: Int J Mol Sci. 2020 Dec 9;21(24):9379. doi: 10.3390/ijms21249379 (PMC7763521; doi:10.3390/ijms21249379)
Supplement: Supplementary file 1 [file ijms-21-09379-s001.pdf]

Figure S1. Immunofluorescence staining of CK18 and Igκ in primary hepatocytes.

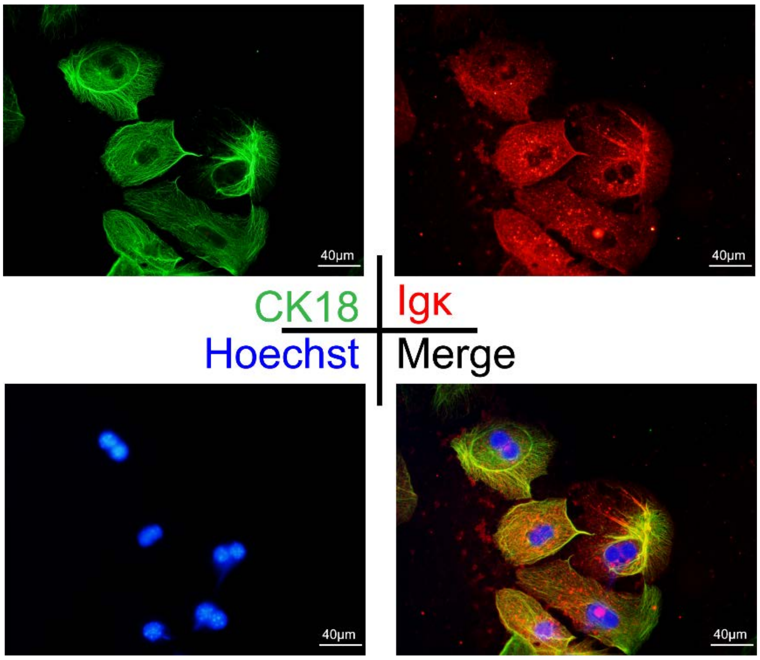

Figure S1. Immunofluorescence staining of CK18 and Igκ in primary hepatocytes.

Figure S2. Hepatocyte-derived Igκ inhibited ConA-induced liver injury *in vivo.*

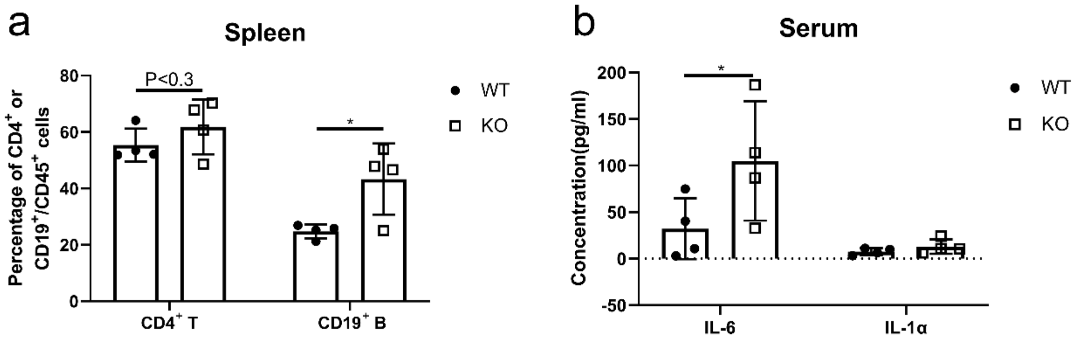

Figure S2. Hepatocyte-derived Igκ inhibited ConA-induced liver injury *in vivo.* WT and KO mice were stimulated by PBS or ConA for 24h and sacrificed 24 h later. **(a)** Percentage of spleen immune cell subsets was analyzed. (**b)** The concentrations of serum inflammatory factors in WT and KO mice were measured, $p<0.05$.

Figure S3. Knockout of *Igκ* promoted hepatocytes apoptosis after ConA or LPS treatment *in vitro.*

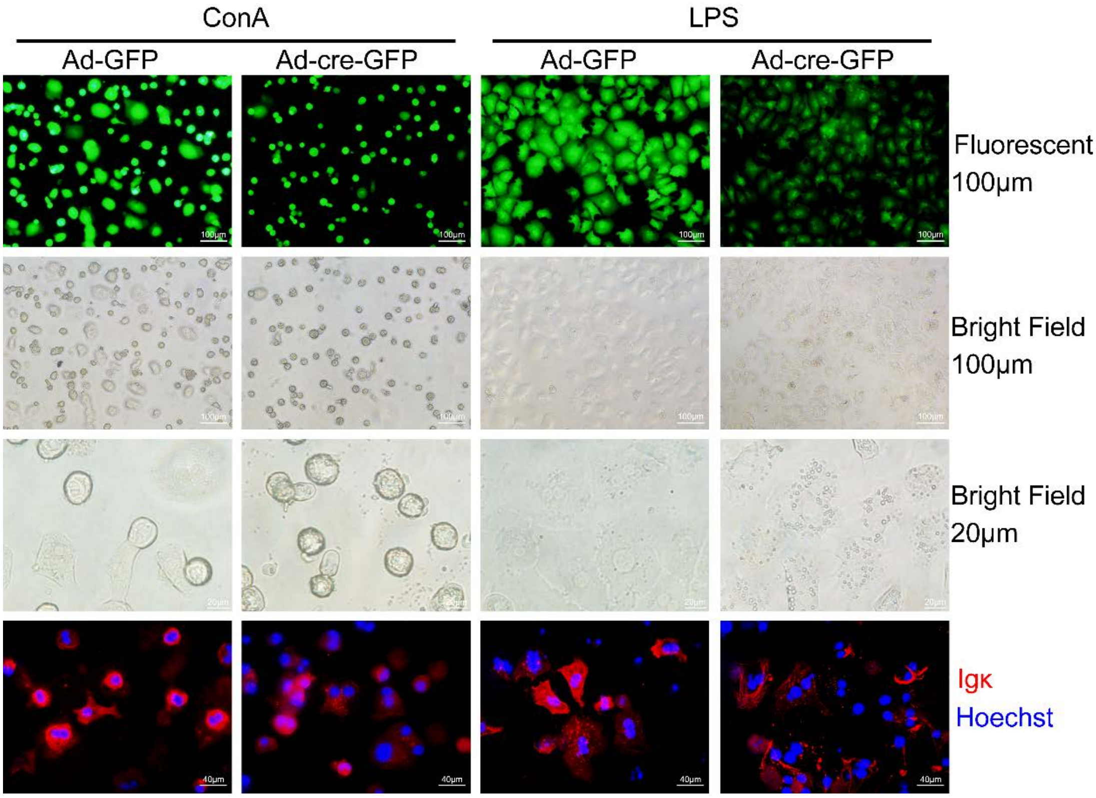

Figure S3. Knockout of *Igκ* promoted hepatocytes apoptosis after ConA or LPS treatment *in vitro.* Primary hepatocytes isolated from *Igκ*$^{fl/fl}$ mice were stimulated with concanavalin A and lipopolysaccharide after Ad-cre-GFP or Ad-GFP infection for 24 h. Fluorescent and bright-field images of primary hepatocytes were shown, and immunofluorescence staining of Igκ was detected.
